# New forms of development: branding innovative ideas and bidding for foreign aid in the maternal and child health service in Nepal

**DOI:** 10.1186/s12992-018-0350-0

**Published:** 2018-03-27

**Authors:** Radha Adhikari, Pam Smith, Jeevan Raj Sharma, Obindra Bahadur Chand

**Affiliations:** 1 Nursing Studies, School of Health in Social Science, University of Edinburgh, Doorway 6, Teviot Place, Edinburgh, EH8 9AG UK; 2 School of Social and Political Science, University of Edinburgh, Crystal Macmillan Building, 15a, George Square, Edinburgh, EH8 9LD UK; 3 Social Science Baha, 345, Ramchandra Marga, Battisputali, Kathmandu-9, Nepal

**Keywords:** Foreign aid, Maternal and child health, Intermediary Organisations, Low-income countries, Nepal, Health service development

## Abstract

**Background:**

Nepal has been receiving foreign aid since the early 1950s. Currently, the country’s health care system is heavily dependent on aid, even for the provision of basic health services to its people. Globally, the mechanism for the dispersal of foreign aid is becoming increasingly complex. Numerous stakeholders are involved at various levels: donors, intermediary organisations, project-implementing partners and the beneficiaries, engaging not only in Nepal but also globally. To illustrate how branding and bidding occurs, and to discuss how this process has become increasingly vital in securing foreign aid to run MCH activities in Nepal.

**Method:**

This paper is based on a qualitative study. The data collection method includes Key Informant Interviews, the review of relevant policy documents and secondary data, and finally field observation visits to four maternal and child health (MCH) projects, currently funded by foreign aid. Through these methods we planned to gain a comprehensive understanding of the aid dispersing mechanism, and the aid-securing strategies, used by organisations seeking funds to provide MCH services in Nepal.

**Results:**

Study findings suggest that foreign aid for the provision of MCH services in Nepal is channeled increasingly to its beneficiaries, not through the Government system, but rather via various intermediary organisations, employing branding and bidding processes. These organisations adapt commercial models, seeking to justify their ‘cost-effectiveness’. They argue that they are ‘yielding good value for money’, with short-term target oriented projects. This ethos is evident throughout the aid dispersing chain. Organisations use innovative ideas and intervention packages, branded internationally and nationally, and employ the appropriate language of commerce in their bid to secure funds. The paper raises an important question as to whether the current mechanisms of channeling foreign aid in the MCH sector, via intermediary organisations, can actually be cost-effective, given the complex bureaucratic processes involved.

**Conclusions:**

The study findings are very important, for Nepal’s development in particular, and for international development in general. The paper concludes by recommending strongly that foreign aid should concentrate on supporting and strengthening the national government system. Complex bureaucratic process must be minimised and streamlined in order to provide quality care to the beneficiaries.

## Introduction

Currently, foreign aid in health, particularly in the maternal and child health (MCH) sector, occupies a significant socio-political space globally. The industry is huge.^1^ Its business is complex, as numerous stakeholders are involved at various levels: donors, intermediary organizations, MCH project-implementing partners and the beneficiaries; all engaging at international, national and local levels and fulfilling diverse roles.

Since the early 1950s, after the Second World War, foreign aid for international development has undergone many changes with regards to its dispersal mechanisms, institutional arrangements, and indeed the language around these issues. In recent decades, in order to get ‘good value for money’ and make aid ‘effective’, funding is increasingly channeled through various intermediary organisations. In this paper we draw on our findings to discuss only one aspect of foreign aid. We focus on how some of the organisations, involved in the MCH sector in Nepal, source aid-funded project contracts, by a competitive process of branding intervention packages and bidding practices.

In this paper ‘branding’ means the development of innovative MCH service intervention ideas, or creating an identity of a particular aspect of MCH service intervention. Either this method is designed to operate at national and global levels or it involves bringing together seemingly cost effective MCH intervention packages. These may comprise ideas, modes of engagement, targets, and time bound goals. Once presented to both donors and policy audiences, they are then promoted. The process requires the support of expert professionals and organisations working in the MCH sector. Branded MCH intervention packages should have a good track record, and the professionals involved should have considerable expertise in their use, as they can have a major impact on improving mothers’ and babies’ health. The term ‘bidding’ means using or marketing those ideas, whilst going through the requisite competitive selection processes to secure funds to run projects. The national and global use of branding ideas and intervention packages, and bidding for project contracts, is the primary focus of this paper.

## Background

Nepal has been receiving foreign aid and technical assistance in health sector development, and that of other sectors, as noted above, since the early 1950s, at the time of gaining its first multiparty democracy [1–5]. It began by receiving foreign aid from neighbouring countries in Asia: namely India and China. Affluent countries, such as the United Kingdom (UK), the United States of America (USA), Japan and Russia (formerly the USSR), amongst others, followed. The first reported foreign aid to enter Nepal, in an organised fashion, was from the USA and from India [2, 6, 7].^2^

For the past sixty years, foreign aid has been making a consistent and significant contribution to Nepal’s health sector, including the MCH sector [6–8]. Foreign aid has been crucial to national health policymaking and health system capacity building. It has supported health professional education and health service provision nationally. Currently, the main foreign aid contributors to Nepal include bilateral donors (USAID, UK aid and Japanese aid) and multi-lateral donors (UNICEF, World Bank, World Health Organisation, Asian Development Bank). Other major sources of aid are from international non-governmental and charitable and humanitarian organisations, such as the United Mission to Nepal and the International Red Cross.

As Nepal (in the 90s) was one of the countries with unacceptably high maternal and child mortality rates, it became the focus for a number of international donors, including UK UKaid, and USAID. In order to improve MCH statistics, there was an urgent need to strengthen the country’s under-resourced MCH services. Foreign aid and an on-going commitment made by international donors, allied to the Government of Nepal’s own commitment towards achieving the Millennium Development Goals (MDGs), have been crucial to the successes achieved in this field. Indeed Nepal’s considerable progress has been presented globally as a success story [9, 10]. Foreign aid support has been shown to be key to a nation’s development and its health service provision, not only for Nepal, but also for other low-income countries.

Whilst foreign aid has been vital for the functioning of health systems in many low-income countries, it has also created some contentious debate [11]. These are mainly surrounding issues of ‘aid effectiveness’, ‘value for money’, its possibly ‘fuelling corruption’ and increasing some countries’ ‘dependency on aid’ [12–14]. There is much debate too around its potential to be a ‘remedy for the world’s poverty and suffering’ [15].

With maternal and child health being a priority area, there was a global health policy and funding shift to meet specific targets related to MDGs Four and Five. ‘Cost effectiveness’ of any intervention was of further importance. In order to achieve this, most donors and intermediary organisations routinely use non-governmental organisations (NGOs), or private sector service providers, to carry out functions relating to health service development and delivery, both nationally and internationally [15, 16]. These intermediaries include non-profit organisations, private contractors, management consultancies, advocacy groups, research organisations, think tanks and educational institutions. They occupy and link the space between the funders and the beneficiaries or target groups, translating the meanings and processes of development to both parties [17, 18].

Scholars and professionals, working in the foreign aid and international development field, have chronicled the various stages in the modern-day history of foreign aid, as it has been influenced by various global political and economic situations [2, 19, 20]. In the 1980s, major international financial institutions, such as the International Monetary Fund and the World Bank, created various conditions, the so-called Structural Adjustment Programme, to aid recipient low-income countries when receiving international aid [19]. The Paris Declaration of 2005 was another major turning point for global aid policy direction. It seems to have shaped present-day aid-channeling mechanisms, with their focus on ‘value for money’, ‘accountability’ and ‘transparency’. In the global context of foreign aid in the MCH sector, ‘value for money’ and ‘aid effectiveness’ are the most discussed topics of the twenty-first century [18, 20–22]. Against this backdrop, this paper will discuss foreign aid dispersing and securing practices in Nepal in general, and common foreign-aid sourcing strategies used by national and local non-governmental applicant organisations, working in the field of maternal and child health.

## Method

This paper is based on a qualitative study that aimed to generate policy-relevant knowledge on the roles and functions of foreign aid related institutions, be they donors, intermediaries or local implementing partners, in MCH service development and delivery in Nepal and Malawi. The two countries were selected because they share similarities in their health sector and socio-political contexts. Both are heavily aid-dependent and are amongst the poorest in the world, in terms of their economic ranking and poor maternal and child health statistics. The study is extensive. It looks at the similarities and differences between the two countries and at the global implications of these findings. However, the data presented in this paper is directly related to foreign aid sourcing and channeling mechanisms, as regards MCH service provision in Nepal.

### Fieldwork and data collection processes

Data were collected using a number of qualitative research approaches. We began with a stakeholder mapping exercise, for which we had developed guidelines. We charted all foreign aid funded MCH projects, working in Nepal from the 1990s till 2016, either via the Government system or out with it. We reviewed all available data sets, policy documents, literature, and also pursued word of mouth recommendations. Having compiled a list of stakeholders involved in MCH, we ran an inception workshop. We invited people from key stakeholder organizations, identified as donors or intermediaries, be they international, national or local implementing partners in the country. This facilitated our entry to the field, and allowed us to gain a broader understanding of foreign aid and MCH services, and to verify and update the list of stakeholders.

From the list of stakeholders involved in MCH, we selected four projects for in-depth study. Selection criteria here were: 1) organisations working in the MCH field, 2) those funded by foreign aid, and 3) a willingness to participate in the study. We also wanted to capture the diversity of organisations, and include large and small-scale funders, long and short-term projects, and projects working in different geographical locations in Nepal. The four case study projects in Nepal were: Aama Surachaya (embedded within the Nepal government system and supported by multiple foreign donors); Suaahara phase-1 (USAID funded and managed by a consortium, led by Save the Children Nepal); Strengthening Maternal Child and Reproductive Health or SAMMAN (Glaxo-Smith Kline funded and Care Nepal managed) and finally Strengthening Reproductive Health or SRH (Japanese Government funded and Adventist Development Relief Agency [ADRA] Japan and ADRA Nepal managed).

After selection we visited all four project implementing field sites, spending time with field staff to gain a deeper understanding as to how these projects were run.^3^ We interviewed staff members in each project, with a particular focus on how they receive foreign aid, and how the money is channeled thereafter to carry out MCH activities. In total we conducted 39 Key Informant Interviews (KIIs) in Nepal. These were of government policy makers, donors, and independent consultants working in the MCH sector and of staff working in intermediary organisations.

During our visits to case study sites in various districts we kept field notes, recording all key observations we made on the everyday functioning of the MCH projects. We also made notes of any related activities, such as education on nutrition and a balanced diet and resources available in the MCH clinics.

Undertaking this in-depth case study, of four foreign-aid funded MCH projects, has allowed us to produce detailed data on current bidding and branding practices, donor behaviours and the bidding strategies used by intermediary, and service implementing organisations, in Nepal. As such, information gathered in this study has been the result of long-term engagement with major stakeholders involved in MCH service provision in Nepal. These have included foreign aid donors, staff members in the Government of Nepal health systems, and intermediaries and beneficiaries of current MCH projects. Furthermore, members of the research team have been working in Nepal’s health sector for over two decades, and so have good relationships with MCH professionals and in-depth insights as to how the sector works.

### Data analysis

All 39 interviews (KIIs) were digitally recorded, and were later transcribed and translated. Our field observation notes too were recorded, and we read and re-read our notes thereafter to analyse data. We also analysed relevant policy documents and media coverage on foreign aid and MCH in Nepal. We held monthly meetings, during which emerging themes were identified. We discussed our progress in the data-gathering process and, in addition to routine discussions and meetings, we organised a two-day mid-term workshop as a further check of our progress. At this time we systematically discussed all data gathered from each of our case study projects, including KIIs in detail. All six members of the Nepal-based research team participated in data analysis, and information was verified and crosschecked by each member for its validity.

### Ethical consideration

Ethical clearance was obtained from the Nepal Health Research Council. We also followed Economic and Social Research Council (ESRC)/ Department for International Development (DFID), the funder’s ethical guidelines, and those of the university, which housed this study. Informed consent was obtained from all individuals and institutions involved. Apart from the information that was in the public sphere, individual informants were anonymised to protect their identity.

## Results

Findings from this study suggest that foreign aid enters Nepal via a number of routes. Some donors provide direct support to the government of Nepal to carry out service and development activities in the country. These are known as pool donors, with funding going through the government system. Other (non-pool) donors favour NGOs, or international non-governmental organisations (INGOs), to disperse the funding to the beneficiaries. A wide range of donors is contributing to MCH service provision, and to other development activities, within the country. Donors may be bilateral or multilateral. Religious and charitable organisations, private individuals and companies are all contributors. Some of these are long-established donors and some more recent.

The route the funding takes depends upon the donors’ values, their relationships with the recipient organisations and their strategic interests. Whichever route the funding takes, and whatever forms it comes in, there are some common, but also regularly shifting, trends and patterns around foreign aid channeling mechanisms. However, a key point seems to be that most foreign aid donors want to maximise the benefits, by making aid cost-effective and value for money. Both aspects are therefore very important current funding criteria.

Understanding current foreign aid trends and donors’ strategic interests, and then following them, is evidently key for all applicant organisations in their bid to secure funds. MCH service-implementing partners and intermediary organisations, at national, regional or local levels, and those who wish to obtain foreign aid strategically, consider these factors, while preparing for funding applications. This study has identified some common trends currently practised in Nepal. These are the *branding of innovative MCH ideas and intervention packages and the promoting of them while bidding for funding.* These strategies and ideas are now further explored.

### Branding of innovative development ideas and MCH service intervention packages

The commercial branding of products, such as Coca-cola or Nike, is commonplace today, but the idea of branding seems to be penetrating the modern global healthcare system, and indeed beginning to dominate it. From the ethnography of our research case study projects and KIIs, one important common finding emerges: all stakeholders involved in MCH services in Nepal use branding and the promotion of their innovative development ideas and intervention packages. Moreover, the majority of foreign aid donors, intermediary organisations and local implementing partners appear to fully engage in branding and bidding processes.^4^

Branding begins with developing a new and innovative idea for a MCH service intervention, examples of which are listed in Table 1 below. From our observations during fieldwork, we learned that these ideas and interventions seek to be ‘unique’ and preferably ‘tested and tried with a good track record’. If these ideas have been tried elsewhere before, they are ‘adapted or scaled up’ in Nepal. Old ideas, such as ‘multi-sectoral approach’, and ‘neo-natal resuscitation’ are given new names to make them sound more innovative and cost-effective. They are made to seem more relevant to the local context, to have the potential of achieving a good ‘impact’ and to be ‘good value for money’.^5^ This latter can also be about gaining social capital.^6^ We further learned that some of these innovative ideas and intervention packages are piloted and scaled-up nationally in Nepal, and are then implemented in other low-income countries with a similar socio-political context, and vice versa. The more seemingly innovative and cost-effective ideas the bidder presents, the more likely they are to be successful in the bidding process and to secure funding.

In addition to this, the bidder organisations need to show that they have the capacity, and are best positioned institutionally, to carry out the project activities effectively. This is where the role of NGOs, as brokers or intermediaries, comes into play, linking the local community with global policies. As such, foreign aid is dispersed increasingly by ‘competitive bidding’ practices.^7^ MCH is only one example, for the process of branding and bidding we witnessed is common in many other sectors of development in Nepal, including education, literacy, and agriculture.

Organisations involved in MCH service provision and the branding of ideas and interventions function at various levels: internationally, nationally and locally. During this study we also encountered some globally branded MCH ideas and intervention packages implemented nationally and locally in Nepal. These are copy-written by the programme developers, and distributed and sold as training and service packages. Organisations wishing to use one of these globally branded programmes are required therefore to buy them.

**Table 1:** Some examples of MCH organizational brands used in case study projects in Nepal (and internationally)

MCH organization	Programme	Branded ideas/interventions	Scope
Care Nepal	Strengthening Maternal Child and Reproductive Health (SAMMAN)- Case study	Community Health Score Board (CHSB)	National level programme (in 2016)
Consortium to run Suaahara Phase −1 in Nepal	Suaahara Phase-1 – case study	Multi-sector approach	National level programme (in 2016)
Adventist Development Relief Agency (Japan)	Strengthening Reproductive Health (SRH) – case study	Hardware and Software Support	National level programme (in 2016)
American Academy of Pediatrics	Implementing partners globally	Helping Babies Breathe and Helping Mothers Survive –Bleeding After Birth	Global programme, implemented in Nepal too.
Currently the Government of Nepal	Aama Surachays (evolved from safer motherhood programme) – case study	Maternity Incentives and many other features	National programme in Nepal and similar programmes are implemented in many low-income countries
Tuki	SUAAHARA’s implementing partner	A tool called MEAL – Monitoring, Evaluation, Accountability and Learning	District level implementing partner organization (in 2016)

The aim of this table is to illustrate some of the organisations involved in the MCH sector in Nepal and indicate some of the innovative ideas and branded intervention packages they use. It does not provide a complete list

One such example is the Helping Babies Breathe (HBB) training package. A global programme, designed to meet MDG Four, it focuses specifically on preventing neonatal asphyxia, considered a leading cause of neonatal death. The major component of the programme is capacity building, through equipping health workers, particularly birth attendants, with the necessary skills in, and knowledge of, neonatal resuscitation. By providing them with training on how to take care of babies immediately after birth, and through supplying them with the basic equipment to carry out set HBB-related activities, it is hoped to reduce neonatal deaths. The HBB official website states^8^:

… HBB is an evidence-based educational program to teach neonatal resuscitation techniques in resource-limited areas. It is an initiative of the American Academy of Pediatrics (AAP) in collaboration with the World Health Organization (WHO), US Agency for International Development (USAID), Saving Newborn Lives, the National Institute of Child Health and Development, and a number of other global health organizations….

Released in June 2010, for application mainly in low-income countries, the HBB programme was rolled out in December 2011 to 34 countries and, by April 2013, this number had increased to 59 countries.

Currently, there are many similar examples of globally branded training packages in the MCH field, widely promoted across many low-income countries, including Nepal. These include Helping Mothers Survive-Bleeding After Birth (HMS-BAB), Practical Obstetric Multi-professional Training (PROMPT: branded and promoted by the PROMPT Maternity Foundation) and Kangaroo Mother care.^9^

National and local organisations involved in the MCH sectors have developed their own national or local brands. One such example is Care Nepal, an INGO working in Nepal since 1978, whose tool kit, the Community Health Score Board (CHSB), assesses and measures the progress of health interventions in districts. Proud of its CHSB, its staff informed us during our field visit that, through use of this very innovative tool, project outcomes could be measured properly and accurately. Further claims for this tool are that is very cost effective and easy to use, and that it is unique to Nepal. In early 2016, this tool was used in 51 districts in the health field, and was also adapted and introduced to the forestry sector. During this study, in 2014–16, Care Nepal actively promoted CHSB when bidding for funding, presenting it to the funder as its organisational strength.^10^

In similar ways, three other case study organisations (listed in Table 1), involved in MCH service provision in Nepal, have developed their own branded ideas, and are involved in using and promoting what they see as their unique brand. One of these is^11^:

…Suaahara, for instance is the project of such a kind in Nepal. This project not only focuses on maternal and child health but also has integrated nutrition, agriculture, family planning, water and sanitation within it….

At a national level, Suaahara promotes a ‘multi-sectorial’ approach to improve maternal and child health in Nepal. Banners and billboards promoting Suaahara’s activities can be seen in all project-implementing districts countrywide. On numerous occasions we were reminded of how innovative this approach is, how very cost effective it is, and of its potential to have a long-term impact in the reduction of maternal and child morbidity and mortality.

Aama Surakshya, the maternity incentive scheme, is a further example of an initiative seeking to make a difference. This scheme appears to be a new improved version of the Safer Motherhood Initiative. It claims that it contributes to better mother and child health through offering incentives to promote institutional birth, and supplying a *Nyano Jhola* (warm bag) to post-natal mothers. The Maternity Incentive Scheme under the Safer Motherhood banner has however, currently become a global brand. Piloted in Nepal in 2005 in a few districts, it had been found to be an effective way to save mothers’ and babies’ lives and to reduce maternal and child mortality, by encouraging women to give birth within an institution.

We found many examples of organisations claiming their ideas are a special brand, and their approach constitutes the area of expertise in the MCH field. ADRA Nepal, a charity organisation working in the MCH sector, claims it provides ‘hardware’ and ‘software’ support to rural health facilities, ensuring MCH service provision in order to save women’s and children’s lives.^12^ It frequently promotes its ‘hardware’ and ‘software’ support ideas, alongside its sustainability and expertise in capacity building work in the MCH sector. It is clear that most organisations seem to have some sort of branding of their ideas and intervention packages.

Within the wider context of branding development ideas and initiatives, a district level NGO, called ‘Tuki’, based in Sindhupalchowk (east of Kathmandu valley), has adapted a project monitoring and evaluation tool. Branded globally as MEAL (Monitoring, Evaluation Accountability and Learning), it has been promoted locally as Tuki’s strength, thereby earning significant social capital. We were informed about it thus^13^:

…Tuki uses principle of **MEAL** system: Monitoring Evaluation Accountability and Learning. All activities have to be very transparent. There is a free contact number for public to contact Tuki for further enquiry….

We were further informed about Tuki’s expertise in using MEAL, its being a system ensuring value for money and transparency, and there being no space for an organisation to misuse funds and other resources. NGOs strengthen their bids to secure further funding by their use of such ‘proven’ methods.

### Promoting of the branded MCH ideas and intervention packages while bidding for funding

Promoting the new ideas and bidding for funding are the next steps. These are again conducted at global, national and local levels. Once the ideas and intervention packages are ready for implementation, and the producers have ensured any necessary copyrighting and patenting is in place, then promotion begins in earnest. The aim is not always to seek financial gain, but to earn social capital: trust and recognition. Through raising their organisational profile and track record they may enhance their chances of securing foreign aid. The bidding process is the next step towards their goal.

We have learned that bidding also occurs at different levels, at almost every single stage of the journey: from obtaining funding from major international donors, such as USAID or UK aid, to implementing a MCH project in the community. There are a number of intermediary organisations involved in the bidding, or in the funds channeling processes. Within these groups, some form consortiums and others bid independently. We witnessed two major bids for funding during the study period, both connected with our in-country case study projects, and we will turn now to examine the process more closely.

A consortium of seven different organisations ran Suaahara phase-1, a five-year project funded by USAID. Begun in Spring 2011 it was completed in the Spring of 2016, after which the consortium was disbanded. A close bidding process for Suaahara phase-2, also funded by USAID, was conducted in early 2016, during our study. We learned there were three major applicant consortiums involved, and observed the dynamics of consortium formation and bidding to appear to be pragmatic and fluid.

New consortiums were formed to bid afresh for Suaahara phase-2. Those individual and professional partners and institutions within the consortiums, possessing complementary expertise, or with innovative and branded ideas, and a good track record of using those innovative packages, were perceived to be in a stronger position to secure funding. Helen Keller International, an INGO, was one of the major partners in Suaahara phase-1, but it went on to form a separate alliance with different partner organisations, working in health sector development in Nepal. In between Suaahara phase-1 and phase-2, competitors became friends (or colleagues) and then they became competitors again. At the time of writing, a different consortium is managing Suaahara Phase-2. Led by Helen Keller International, it has been implemented in many new districts in Nepal.

The new consortium formed to bid for Suaahara phase-2, only acted as an intermediary organisation, as in Suaahara phase-1. Suaahara phase-2 received funding from USAID, and then had to find local implementing partners to carry out their activities in implementing districts. All new partners in the consortium brought their own branded development ideas and expertise to the table, and formed new alliances for each new project. ‘Getting value for money’ has been key in all elements of this process: proposal writing, preparing for the project and bidding. Implementing districts are thereafter selected in consultation with the government of Nepal. Table 2 below illustrates how funding for MCH projects was channeled in Nepal in 2014–16.

**Table 2:** Case study project and their fund channeling mechanisms

	Suaahara phase - 1	Strengthening Maternal Child and Reproductive Health (SAMMAN)	Aama Surachaya	SRH
Donor	USAID	GSK	DFID (Technical Assistance),	Government of Japan
Intermediary level − 1	A consortium led by Save the Children Nepal (NGOs)	Care Nepal (NGO)	Government of Nepal	ADRA Japan (INGO)
Intermediary 2				ADRA Nepal (NGO)
Implementing organisation	District level (NGO) in all 41 districts	District level (NGOs) in implementing districts	Within the government system in all implementing districts	Implementing partner (NGOs) in districts

A further case study project we observed was that of SAMMAN. Run by Care Nepal its original locus was in the far-west Nepal. In Spring 2016, the SAMMAN Programme moved to central Nepal and operated in two new districts: Kavrepalanchowk and Sindhuli. In Summer 2016, Care Nepal was engaged in selecting local implementing partners in these districts to run SAMMAN activities. The local implementing partner selection was done, on this occasion, via a bidding process.

### How does bidding work in Nepal?

Big consortia such as Suaahara, a multi-million dollar project, or smaller projects, such as SAMMAN, all go through the bidding process. Intermediary organisations will either receive funds directly from the donors, such as the USAID, or from other bigger international intermediary organisations. They will then channel money to local partners to implement certain aspects of MCH activities in specific districts in Nepal. Here is an example of how Suaahara phase-1 consortium was formed and bidding was initiated^14^:

…While talking further about Suaahara project, Request for Applications (RFA) of the project was announced in early 2011. As we came to know about the announcement of the proposal, we work on the development of the proposal, basically focusing on what will we do? Where are we going to do it? How are we going to implement all of our plans we mentioned? Altogether, we are 7 partners for this project; Save the Children is the main manager for the project. As this project is focused on maternal child health and nutrition, partners are chosen and given responsibility accordingly….

When an intermediary organisation, such as Save the Children Nepal, in alliance with Suaahara phase-1, or with SAMMAN, decides to work in a district, staff members from the intermediary organisation make a preliminary field visit. In the case of an organisation working in health, staff members from that organisation visit the District Public Health Office and liaise closely with the District Public Health Officer (DPHO). In order to select the requisite local implementing partners, a district committee is formed, involving various key stakeholders such as the DPHO and the Local Development Officer amongst others. All local NGOs are invited to submit a letter of interest, before being asked for a full proposal. The committee then draws up a shortlist of potential partners. We were informed that, for the final presentation, the committee usually selects three applicant NGOs working in the district. These will have the strongest proposals, with the most innovative and cost-effective ideas. The shortlisted candidates are asked to give a presentation to the selection committee, outlining their proposed work, organizational strength and budget plans in detail. The Committee will score each organisation, make their final choice and award funds accordingly.

One of our key informants stated^15^:.

…During the bidding process, representatives from bidding organisations are requested to present a detailed future MCH service or intervention plan. At this point the bidders have to perform like sales agents for a commercial company. Funding is granted on the basis of the innovative ideas and strategies bidding that applicant organisations are bringing to the project and how the representative performs at the presentation. Organisational capacity to run a project efficiently is also checked by the donor….

We were informed that this is common practice in Nepal. However, we also learned that there are some exceptions to any perceived rule. Individual and organisational relationships and track records also play their part. We were further informed that, in Suaahara Phase-1, grants were not always awarded to the best-scoring applicants during the application and presentation processes. Before awarding grants, the selection committee staff made a pre-award field visit, to examine the capacity of the bidding organisations to carry out their proposed activities. If committee members were satisfied about the organisational capacity, then the award would be granted, initially for a year.^16^

In the first year, those organisations successful in bidding will have to work on meeting their donor’s criteria, including capacity development if necessary. Bidding processes appeared very similar for most other organisations in Nepal, including the SAMMAN project in Kavrepalanchowk and Sindhuli districts. Intermediary organisations, such as the consortium running Suaahara phase-2, or Care Nepal, will support new partners for a defined period of time. In Suaahara Phase-1’s case for example, the Suaahara district office and their thematic officers^17^ provided all necessary support to the local implementing partners.

After one project finishes intermediary organisations would move on to new districts, and work with new local partners there. From Key Informant Interviews, we learned that a new district or project site selection is made in consultation with Government staff, and new implementing partner selection is conducted via the bidding process. The cycle continues in this way and would appear to be the current, and favoured, form of foreign aid bidding and fund-securing mechanism in Nepal.

### Foreign aid and the MCH post April 2015

A major disaster occurred in Nepal during the course of this study. The tragedy of the April 2015 earthquake was widely broadcast, both nationally and globally. The study team personally witnessed the aftermath of this devastating event, and its impact upon healthcare and other services, both government funded as well as foreign aid funded projects. Healthcare, inclusive of MCH services, was provided in temporary tents. People’s lives were badly affected. The earthquake and the numerous aftershocks created mass panic. All service costs were increased phenomenally. An example of this is described here^18^:.

…In the context of this district, geographical [remoteness] location should be considered as one of the challenges, approximately only 30% VDCs [Village Development Committees] of the district are accessible by road. That is, although some part of the VDCs are accessible to road, not whole area of VDCs, so we have to do on foot monitoring which is very challenging. The geographical area of this district stretches between the very high mountainous area and the Tarai like area, such as Dolalghat. This is one of the geographical challenges. Another is that after the earthquake, costing has also risen drastically. Obviously there are other scarcities as well; let’s not just point out one only. For instance, prior to the earthquake, we paid about 7000 to 8000 thousand [Nepali] rupees for the cost of a vehicle for a monitoring visit. This has now climbed up to 20,000 to 25,000 for the same distance and number of days. The costing of the transportation is now gone up even more than triple than it was before….

However, donors and other foreign aid stakeholders have been flexible in responding to the emergency situation. The competitive branding and bidding process was not applicable while responding to the new MCH service challenges.

## Discussion

The study findings above suggest that, in the field of foreign aid and international development today, be it at local, national or international levels, the branding of ‘cost-effective’ innovative ideas and intervention packages and their promotion are the current norm. It is also clear that ‘competitive bidding’ processes are the favoured method by which to secure foreign aid. In the past few decades, and particularly since the Paris Declaration of 2005, foreign aid in international development has adopted a commercial sector model, in order to yield good value for money, display donor accountability and a transparency of ideas. Bidding organisations now need to have some extraordinary quality, and innovative and cost-effective ideas, and achieve results, which can be presented in numerical forms.^19^ Branding and bidding processes serve the purpose.

Foreign aid policy changes according to international health and development priorities. National and international development actors enter the foreign aid industry stage with new project ideas and innovative packages. For example, in the 1960s and 70s, both in Nepal and internationally, the main focus of foreign aid in the health sector was on an integrated health service. However, since the 1980s, this has shifted to primary health care, with the underlying principles of a multi-sectoral approach and with the aim of providing universal health care by 2000. As discussed above, since the new millennium, the global health service focus has shifted again towards meeting the health-related MDGs (Four, Five and Six). Post the MDG Report of 2015, the new focus is on embracing the idea of ‘Sustainable Development Goals’. During all of these different periods, new ideas and intervention strategies have been developed, implemented, evaluated, scaled-up nationally and adapted internationally. Ideas have then moved on to their next phase, and quite often been sited in different geographical locations. During the study we indeed observed the current focus around foreign aid to be on setting targets, often through short-term projects and, having achieved them, then moving on to different fields.

During the course of this study we witnessed funding calls initiated (advertised in national news media and by other means), bids submitted, and funds secured in ‘competitive’ ways for MCH intervention packages. All were developed with the aim of saving mothers’ and their babies’ lives after birth. It would appear it is not only in the case of MCH services, but also that most other twenty-first century international development projects are very narrowly focused on specific time-bound activities and targets. Measurable interventions are deemed useful therefore, and tangible outcomes are desired. HBB and HMS-BAB programmes ran alongside the improvement of health service provision, in the construction of birthing centres and maternity waiting homes and the training of a targeted number of health professionals, in specific specialist areas. These projects, however, have been worryingly restrictive, short-term and number and statistics focused. In the quest to source foreign aid, the branding of innovative ideas and new interventions packages, which are supported by numbers and statistics, seems to matter the most in any argument justifying cost-effectiveness.

Over time, language in the field of international development changes. As illustrated above new buzzwords, HBB and HMS-BAB to name but two, and innovative ideas (see Table 1), have replaced old ones. All of these factors have caused discourse to shift accordingly. The appropriate use of such words and innovative ideas is crucially important, not only to understand the contemporary language of international development in terms that match the interests of the donors, but also because these words and ideas act as ‘passwords’ to a successful foreign aid application and bidding process [23].

## Conclusion

With shifting global health policies, foreign aid channeling mechanisms are also subject to constant change. However, foreign aid funded projects have been sometimes scaled up and implemented in different districts, with projects terminated in one district after planned targets and goals are met. As illustrated above, the nature of foreign aid funded projects is short-term. For a sustained and quality health service, we argue that foreign aid should focus on supporting and strengthening national government systems in Nepal and other low-income countries. There is plenty of evidence that around 99% of maternal deaths occur in low-income countries with weaker national health systems [24]. Improving the overall standard is key. The focus must be on sustained and quality care in the reduction of maternal and child mortality, not just on branded vertical projects and intervention ideas.

The study, and the writing of this paper, has certainly helped us to understand how foreign aid is secured to run MCH activities in Nepal, to identify the key players involved in the fund channeling process and, finally, to examine the strategies they use to maximise their chances of securing funds. It has also clearly and comprehensively illustrated that there are several layers of intermediaries involved in both stages of the process. Some argue that contracting out has resulted in the giving away of aid to these contractors [15], while others suggest they add new layers to power structures, without replacing pre-existing patronage networks and local centres of power [17].

The process of developing and branding innovative ideas, promoting them and bidding for MCH project requires resources, both in monetary terms as well as in expert personnel. Our impression is that too many resources are being used in branding, bidding and other activities related to all levels of the aid channelling process. As Table 2 above illustrates, there are different layers of foreign aid recipients and sub-recipients. Study findings suggest that a significant amount of time and resources are required in both processes. Findings also generate a few key questions for all stakeholders involved in the channeling of foreign aid for international development. These questions are: Does this new form of development, which basically encourages stakeholders to use often very narrowly vertical and short-term branded ideas and interventions, by creating layers of intermediary players and a supporting bureaucracy, actually yield ‘value for money?’ and ‘Is the process truly cost-effective?’ Based on our findings the evidence appears very doubtful. We strongly feel that it is vital for foreign aid to support and strengthen the national government system and minimise complex bureaucratic processes, in order for it to provide comprehensive, long-term and quality care to the beneficiaries.

## Abbreviations

AAP: American Academy of Pediatrics
ADRA: Adventist Development Relief Agency (Japan)
CHSB: Community Health Score Board
DFID: Department for International Development
DPHO: District Public Health Officer
ESRC: Economic and Social Research Council
HBB: Helping Babies Breath
HMS-BAB: Helping Mother Survive – Bleeding After Birth
INGO: International Non-Governmental Organisation
KII: Key Informant Interview
MCH: Maternal and Child Health
MDG: Millennium Development Goal
MEAL: Monitoring Evaluation Accountability and Learning
NGO: Non-Governmental Organisation
PROMPT: Practical Obstetric Multi-professional Training
SAMMAN: Strengthening Maternal Child and Reproductive Health
SRH: Strengthening Reproductive Health
USAID: US Aid for International Development
WHO: World Health Organisation

## Footnotes

1 We term international development an industry, because of the volume of financial transactions and number of people involved in this field, and the scope of global networks and service provision. There has been a significant increase in total global development assistance in the health sector – from $7 Billion in 1990 to $35.4 billion in 2014. This sector has been frequently described as a foreign aid business and industry by a number of scholars for the same reasons.

2 Globally, the modern idea of foreign aid for international development seems to have emerged mainly after the Second World War. Before this, similar ideas and development activities, in the field of health, education, and overall infrastructure development and service provision existed, usually in countries with weaker economies in Africa and Asia. However, they were not organised in the way we see these today. Pre-WW2 foreign aid support for countries with weaker economies seems to have come from humanitarian and charity agencies, much of it conducted as much to support the existence of the colonial staff as anyone else. It tended to be operated in an opportunistic fashion, and was carried out by religious individuals, organisations and business entrepreneurs (Edwards 2014). Since then, and especially over the past seventy years, the modern idea of foreign aid for international development has evolved. Initially designed to reconstruct war-torn countries in Europe, it has come a long way since then. Currently this sector occupies a significant amount of international socio-political space. It has become one of the most important global industries of the twenty-first century, and one, which continues to evolve rapidly with modern day globalisation.

3 In our study the word ‘project’ refers to a particular activity or activities designed to achieve certain aims and is usually short term. A ‘programme’ denotes a longer-term process, with perhaps multiple stages and the possibility to continue for longer periods with additional funds. Six out of eight MCH case studies (in Nepal and Malawi) are projects and two are programmes. However, in this paper, when we use the term ‘project’, it will also include MCH programmes.

4 Intermediary organisations play key roles in providing funding sources and information to local implementing partners, and information about the local needs and context to the donors. All parties need funding to conduct their business, so become involved in bidding for it. Contracting and sub-contracting out intervention packages is common in the field of international development.

5 The multi-sectoral approach promoted by Suaahara phase-1 is not a new idea in health service development in Nepal. It was much discussed in the 1980s and 90s, and seen as one of the core principles of Primary Health care to achieve the Health for All by 2000 goal. CHECK IF “DOUBLE” QUOTATION MARKS SHOULD BE CHANGED TO ‘SINGLE’.

6 As health professionals in Nepal, we have witnessed the reappearance and recycling of some of these so-called ‘innovative ideas’ around MCH services in Nepal for several decades. However, because of a lack of written evidence, as they were not published, or even documented properly, they do not seem to have had any proven prior existence. In the current day MCH development sector we have noted with interest these same ideas suddenly acquire new names and become the latest innovations.

7 Although we use the term ‘competitive’, we also feel that the process may not be as competitive as it may sound. In practice, bidding and securing funds is often based on individual and institutional relationships.

8
http://www.helpingbabiesbreathe.org/about.html

9 Kangaroo mothercare is also an innovative term, which encourages parents to keep newly born babies as close to their body; maintain skin-to-skin contact and exclusive breast-feeding. Details can be found at: http://www.kangaroomothercare.com

10 Numerous interviews and conversations with Care Nepal field staff during field visits in their programme implementing districts and also in head office in Kathmandu.

11 Interview with the Suaahara Project Manager, August 2014

12 ‘Hardware support’ means providing tangible structural support, such as a building construction and supply of necessary equipment. ‘Software support’ refers to the equipping of health professionals with appropriate knowledge, and supporting them their professional development and skills acquisition, so they are better prepared to provide quality MCH care.

13 Interview with Suaahara District Implementing Officer, 3rd January 2016.

14 Interview with the Suaahara Project Chief, Kathmandu, August 2014.

15 Interview with Suaahara District Implementing Officer, January 2016.

16 Interview with Suaahara District Implementing Officer, January 2016.

17 Thematic Officers are those who are responsible for a specific or themed role. For example, some officers are responsible for water and sanitation whilst others may be responsible for health education and nutrition, or project management, monitoring and evaluation.

18 Interview with the District Project Coordinator, January 2016.

19 Poor MCH statistics, records and numbers can also be seen to be useful while making a funding application. Currently, there is a major global focus on statistics and numbers. In order to be more transparent and accountable to the donors and the beneficiaries, international aid flow needs to be supported by statistics and numbers, as a means to measure aid effectiveness and overall progress. In relation to MCH projects, mortality and morbidity statistics are the basis for granting funds to set up a new project.
